# TOD Typology Based on Urban Renewal: A Classification of Metro Stations for Ningbo City

**DOI:** 10.1007/s40864-021-00153-8

**Published:** 2021-08-12

**Authors:** Liu Yang, Xiaoyu Song

**Affiliations:** China Metro Engineering Consulting Corporation, Building 9#, No.31 Xishiku Dajie, Xicheng Dist., Beijing, 100034 China

**Keywords:** Transit-oriented development, Node-place model, Machine learning, TOD Improvement, Metro, Ningbo

## Abstract

In recent decades, the transit-oriented development (TOD) concept has been widely used all over the world, especially in China, for the massive construction of urban public transportation systems with rail transit as the backbone. However, it is not easy to make significant changes in a city while building a transportation system, and the transit-guided urban development expected by the TOD concept has not been completely realized. The transformation of nearby areas with the guidance of transit is also becoming the choice of many Chinese cities, especially for cities that have only had subways for a few years. Unlike other international metropolitan cities, with metro systems of considerable scale, the modernization process of most of the small and medium-sized cities in China is being carried out simultaneously with metro-based public transportation guidance. For cities which are still in their initial stage of the backbone public transportation system, there is not enough previous experience and evidence to support the suitability of TOD typological analysis based on the node-place model. More research based on the node-place model has also shown practical applications of the TOD in developed cities. However, there are very few studies that analyse cities in which rail transit and urban development are both in a period of rapid growth. The goal of this research is to identify which metro stations in these cities are suitable for TOD improvement and optimization. This article attempts to expand the willingness of residents on the basis of the traditional node-place model as one of the judgment indicators for evaluating whether existing stations and surrounding areas are suitable for TOD improvement. At the same time, traditional statistical analysis is combined with GIS and machine learning technology. Using this method, we propose the TOD improvement-oriented station area classification and identification method based on TOD typology theory. The results show that Ningbo's subway stations can be divided into four categories according to the suitability for TOD improvement, and we selected seven stations suitable for TOD improvement according to the characteristics of the node-place model. The practice in Ningbo has proved that this method is effective for identifying sites suitable for TOD improvement, especially for cities that have recently built subways.

## Introduction

Previously, activities that guided urban renewal and improvement, such as transit-oriented development (TOD), were referred to as joint development [1–3]. Although the activities of transforming cities in this way have a long history [4–7], the concept of TOD was formally proposed by Calthorpe and implied mixed development and a larger population living near transit lines [8]. TOD is widely considered to be an efficient approach for integrating transportation and land use.

China is a country with high population density. Urban extension and the increase in the number of private motorized vehicles have caused traffic congestion, social and spatial integration, and negative environmental, social and economic effects [9]. TOD, as a method for urban planning, has been practised in the populous Asian regions [10–15]. Unlike the urbanized cities in Japan and South Korea, the rapid development of metro systems in mainland China rarely matches the urban land use [16]. Furthermore, the development of stock land will become the mainstream of urban construction in China [17]. With the TOD theoretical input and practical experience gained from other Asian cities, the decision-makers have realized the importance of integrating land development with the transit system, including guiding the development of new towns and constructing commerce around stations [18]. For instance, Ningbo, as a representative city, has formed the idea that ‘building metro is building a city’ to solve the problem of the disconnect between urban development and transportation [19].

More research based on the node-place model has also shown practical applications of TOD in developed cities [14, 20, 21]. However, there are very few studies that analyse cities in which rail transit and urban development are both in a period of rapid growth. The interactive relationship between the establishment of the TOD development model and the implementation of rail transit is often the focus of such cities. This is because these cities have enough opportunities and time to integrate the relationship between urban development and backbone public transportation systems and the land-use patterns guided by them.

This paper attempts to expand the node-place model [22] to assist the urban renewal around the stations. We take the willingness of the residents as an important indicator of pre-planning assessment [20, 23]. In the research, besides using traditional statistical analysis methods, we combine them with GIS and machine learning technology. The advantage of our developed method enables the data model to be less affected by human factors. The proposed model has high repeatability and portability while maintaining the same simple data structure as the traditional model.

The structure of this paper is as follows. Chapter 2 focuses on reviewing the previous node-place model literature and our thoughts on the scalability of the node-place model. Chapter 3 describes the node-place model extension method based on TOD improvement, focusing on the main concepts of the method and the construction method of the index system. Chapter 4 describes the overall calculation process of the Ningbo case, which includes the classification method of TOD improvement based on entropy TOPSIS, machine learning, and other technologies to find the appropriate stations for TOD improvement. Chapter 5 analyses the characteristics and categories of the calculation results, and the recommended stations for improvement. Chapter 6 analyses the situation of the Ningbo metro station based on the model. Finally, Chapter 7 discusses the limitations of the model and the space to be optimized, and expansions for future research direction.

## Literature Review

The quantitative study of TOD began with Bertolini’s research [24] on the development prospect of railway stations and their surrounding areas. Based on the relationship between traffic accessibility and land use, the node-place model was established and successfully applied to the analysis of railway stations in Amsterdam, Utrecht, and Arnhem Nijmegen in the Netherlands [22, 24, 25].

This conceptual model has been quantified and expanded by many researchers to analyse TOD development status assessment [26, 27], traffic behaviour choices [28, 29], land use [30, 31] and many other specific applications. These studies are often attributed to the TOD typology. Although the indicators selected by the model are not the same, the TOD analysis framework based on the node-place model has been widely used. The TOD typologies enhance related planning and design. For instance, the similarities type allows the policymakers and urban planners to develop more targeted sets of strategies to promote TOD [32].

Higgins and Kanaroglou [33] asserted that there are two related approaches to conceptualizing and estimating transit station typologies. The first is to classify according to a certain attribute of TOD, e.g., location of the stations, level of commercial activities, and the function of the transportation (similar to that mentioned by Huang et al. [34]). Calthorpe presupposed that there are three types of TOD in China: commercial centres, urban centres, and town centres [35]. However, it has been discovered through practice that the attributes of TOD areas are diverse, and the Calthorpe approach [35] is unable to describe the multiple attributes of TOD. Dittmar and Poticha [36] also recognized that there can be no ‘one-size-fits-all’ approach to the TOD. The second approach is justified based on the TOD characteristics, which is more positive. Reusser et al. [37] expanded the node-place model and classified over 1600 railway stations in Switzerland into five types characterized by 11 indicators. Chorus and Bertolini [14] used the node-place model to study the traffic types and land-use factors that affect the spatial development around Tokyo’s 99 station areas. Kamruzzaman et al. [32] developed a typology by clustering the stations into TOD-based groups. The analysis in Brisbane showed that the stations might be classified regardless of their balance. Lyu et al. [26] expanded node-place with the ‘oriented’ dimension to quantify the orientation of transit and development components towards each other. The authors determined the accessibility and walking potential by measuring the spatial specifications and walkway density parameters. Because of the particular research objects and the requirements of practical projects, the current study adopts the second typology.

## Methodology

### Theoretical Background: The Node-Place Model

**Fig. 1:**
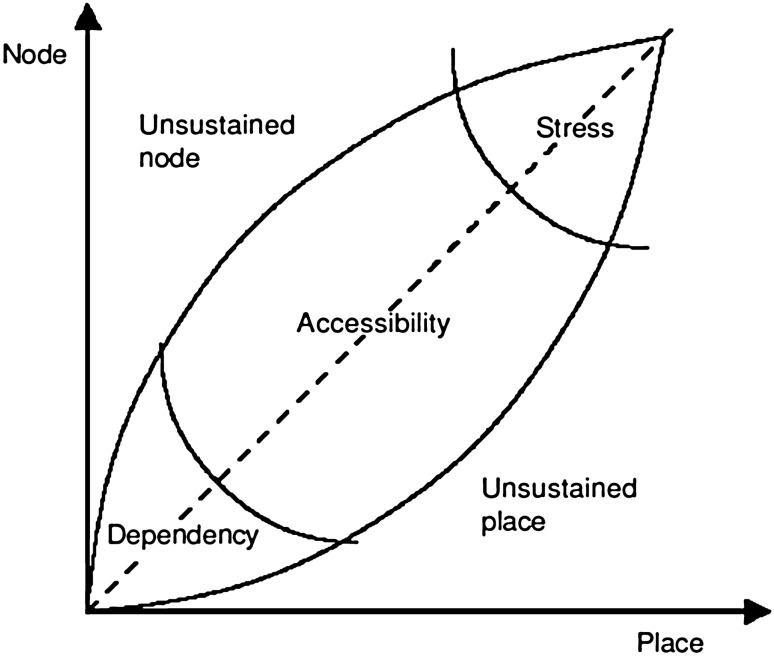
Node-place typology model [22]

The node-place model proposed by Bertolini [22] is an important tool for TOD analysis for public transportation stations (see Fig. 1). The basic idea of the model is to treat the stations as a combination of nodes in the transportation network and places of urban functions [38]. Analysis of the influencing factors attributed to the transport functions (node characteristics) and urban functions (place characteristics) shows that the coordinate positions of the two attributes reflect the role and status of the station and its surrounding areas throughout the city.

According to the coordinate position on the map, all theoretically existing stations are divided into the five following categories:

1. Stress (S area)
2. Unsustained node (U-N area)
3. Accessibility (Ac area)
4. Unsustained place (U-P area)
5. Dependency (D area)

In a specific city, however, there may not be necessarily all five types of stations. Moreover, this conceptual model does not clarify the function expression of each curve and it is only a conceptual model that represents the relative position of the station. In the related research, there are a variety of quantitative methods. Some of these methods use standard values [26, 39], and some perform z-transformation [14, 37]. In addition, the indicators used in various studies are different according to the specific local conditions, including the selection of indicators and the range of specific indicators (such as the difference in the suitable walking ranges). The common feature, however, is that they all fall into two attributes (node, place) or three attributes (T-attribute/node, D-attribute/place and O-attribute).

### Node-Place Model Based on TOD Improvement

**Fig. 2:**
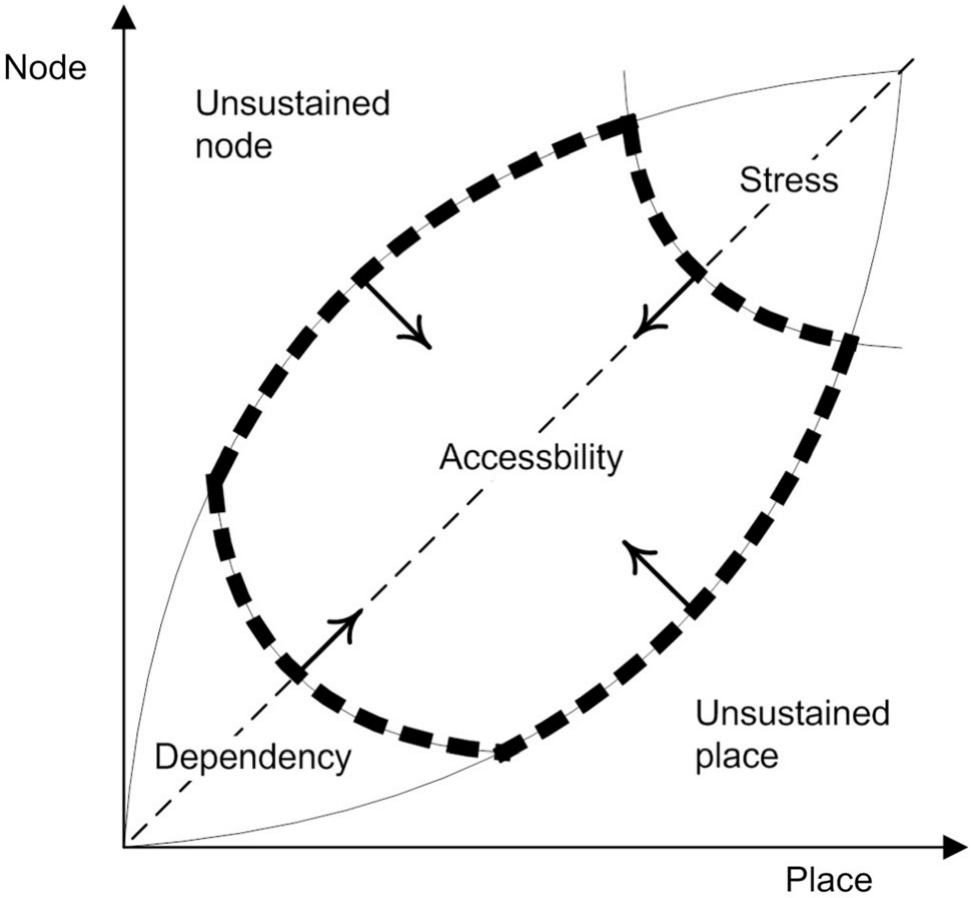
Node-place model based on the TOD improvement (four centripetal arrows show the ideal direction of improvement)

According to the basic node-place model theory, the four categories including stress (S), unsustained node (U-N), unsustained place (U-P), and dependency (D) are not ideal station types. Therefore, when selecting the TOD improvement and optimization object stations, it is considered to select the stations close to the critical state of the four sides of the accessibility (Ac) area, achieving the accessibility (Ac) state as the improvement goal. Note that the specific implementation method also depends on the actual situation of the station. Although some stations have certain TOD development bases, such as population and job density, they have not reached the ideal TOD development level and status. In some cases, areas with dense residential and commercial facilities have not become the regional centre, while in others the commercial core area of the old city has gradually lost its vitality with a geographical centre position (see Fig. 2). Compared with Beijing, Shanghai, Hong Kong and other international cities with more developed and stable rail transits, some ordinary Chinese cities such as Ningbo, with most of the stations having not been developed at all (numerous dependency-type stations tending to the lower-left corner), are selected as the objects of improvement. In such cases, although the station itself may be greatly improved, such stations that have just had metro services are not yet in urbanization or on the edge of urbanization. Therefore, there is no basis for the TOD improvement in the short term. So it is often better to select some stations near the four arcs near the edge of area A to upgrade these stations mainly in area D to area A. This will make it easier for the transformation to take effect, and the cost is lower.

### TOD Improvement Site Identification Method for Entropy TOPSIS Based on the Node-Place Model

Measuring station areas worthy of TOD improvement is similar to TOD development level evaluation [32, 37, 40] and station classification [26, 27, 29, 39]. It is necessary to carry out a quantitative analysis for the influencing factors of multiple dimensions and further calculate according to the method of multi-criteria decision analysis (MCDA) [18, 41, 42]. Considering that the human factors often interfere with the objectivity of the calculation results, we use the entropy TOPSIS (E-TOPSIS) [43] for the traditional MCDA method. The scores that each station receives by E-TOPSIS analysis reflect the current TOD development level and the basic value of TOD improvement typology. Therefore, besides the description of general transport, density, urban design, etc., the indicator system should also include metro passengers’ attitudes (i.e., their revealed preference) and the wishes of potential metro passengers (i.e., their stated preference).

At the same time, these factors should be associated with one of the T/O/D attributes, so that the entire index system can also obtain the calculation results of each station in the T/O/D three-dimensional coordinate system based on the node-place model. Therefore, the node-place model combined with the E-TOPSIS calculation results can be used to screen out the stations that have a certain TOD development basis and improvement value.

### Principles of TOD Improvement Indicator Selection

There have been many studies discussing the selection of TOD indicators based on the node-place model [14, 26, 27, 29, 32, 39, 44]. Based on the above results of systematic research, there are six common principles as follows.

- Follow the TOD principle; the most famous is the 3D (density, diversity, and design) principle proposed by Cervero [45]. Cervero et al. then extended the theory to 5D [46] (with the extra dimensions including Destination accessibility and Distance to transit), and later 7D [47] (with the extra dimensions including demand management and demographics). In any case, the 3D principle as the core foundation of TOD is the general foundation.
- Reflect the characteristics of their respective cities. Chinese cities are undergoing rapid development and changes in recent years [48], especially the rapid growth of the backbone public transportation systems, e.g., rail transit and bus rapid transit (BRT). Therefore, TOD in China is related more to public transportation and urban planning [16, 49, 50].
- Focus on the middle and micro features around the station. In collating the related studies, it can be found that the social constituent factors and economic service characteristics are the fundamental aspects that reflect the development level around the stations.
- As a task with TOD improvement and its value orientation, it is necessary to survey the public regarding their preferences, although such surveys are often affected by the actual social conditions in various regions [20, 23].
- The index system has universal applicability. The broad attributes of the index system should allow direct comparison with those of other cities and related research. The weighting of the indicators should therefore minimize subjective judgments.
- The calculated result of the index reflects the direction to be transformed for the reference of the implementing entity. Further, the specific indicators can be appropriately modified according to the needs of the project, if the value orientation of the project is reflected.

### Identification of the TOD Indicators

Based on the above principles and regarding the related research, we selected 39 indicators within eight categories covering the three dimensions of T/O/D, as shown in Table 1. According to the 3D principle [45] and collected data from Ningbo local planning institute, the population density, the job density and the floor area ratio as three types of data for the nine indicators (all of them including the data of the three layers with radii of 250 m, 500 m, and 1000 m) are more suitable as indicators for the selected items. According to the mix, and the principle of land use [30, 44, 51], we use entropy to calculate the diversity of land properties. We further introduce the rationality index of the circle development to describe the different radii around the station change trend of floor area ratio. Among the factors of urban design combined with the needs for TOD improvement, we select the indicators related to the development potential and transportation connection.

**Table 1:** Criteria and Indicators for measuring TOD index

Attributes	C. no.	Criteria	I. no.	Indicator
Transit	1	Rail transit (T1)	1	Inbound passenger flow
			2	Outbound passenger flow
			3	Number of station entrances and exits
			4	Connecting passenger flow (0–500 m)
			5	Connecting passenger flow between (500–1500 m)
			6	Connecting passenger flow (1500 m+)
	2	Traffic connection (T2)	7	The convenience of connection (CCI)
			8	Number of intersections
			9	Number of bus stations
			10	Number of bus routes
			11	The density of the road network suitable for walking
			12	The density of the road network suitable for bicycles
			13	If there are parking lots for non-motor vehicles
			14	If there are P+R parking lots
Oriented	3	Residents willingness (O1)	15	Residents' daily living and travel locations
			16	Commercial consumption areas
			17	Metro passengers' willingness to transform
			18	Non-metro passengers' willingness to take the metro
	4	Development potential around the station (O2)	19	Commercial development potential
			20	Public space connectivity
			21	If there is unbuilt land
Development	5	Density (D1)	22	Population density (0–250 m)
			23	Population density (250–500 m)
			24	Population density (500–1000 m)
			25	Job density (0–250 m)
			26	Job density (250–500 m)
			27	Job density (500–1000 m)
	6	Diversity (land use) (D2)	28	Floor area ratio (0–250 m)
			29	Floor area ratio (250–500 m)
			30	Floor area ratio (500–1000 m)
			31	The entropy of the land properties
			32	Rationality index of circle development (Standard Gradient Index)
	7	Social service(D3)	33	Density of restaurants
			34	Density of convenience stores
			35	Density of cultural and sports facilities
			36	Density of medical facilities
			37	Density of residential quarters
	8	Economic development(D4)	38	Residential prices
			39	Commercial rents

The development potential criteria consist of three indicators: commercial development potential, public space connectivity, and presence of unbuilt land. Based on related research on traffic connection criteria [47, 50, 52, 53], three indicators are selected to describe the features of this aspect: the number of intersections, the density of the road network suitable for walking, and the density of the road network suitable for bicycles. The convenience of the connection is then described using the current average connection distance of the passenger connected to the subway. The design of the transportation connection facilities criteria is based on four indicators: the number of bus stations, the number of bus routes, whether there are parking lots for non-motor vehicles and P+R parking lots.

The selection of traffic indicators is site-specific. The Ningbo metro has only been opened to traffic for 6 years, the network is not complicated, the vehicle type selection is relatively simple, and the difference in line capacity is insignificant. Therefore, six indicators are selected to describe the traffic criteria including the inbound passenger flow, the outbound passenger flow, the number of station entrances and exits, and the connecting passenger flow of the three circles below 500 m, 500–1000 m and beyond 1000 m.

Regarding the social services and economic development criteria, we refer to the related research [18, 44, 54] combined with the actual situation in Ningbo. The selected indicators are the density of restaurants, convenience stores, cultural and sports facilities, medical facilities, and residential quarters. In addition, residential prices and commercial rents are also selected as indicators of the economic status of the station area. Similar studies [18, 44] also mentioned that the setting of indicators should have a certain level of flexibility to be able to incorporate the differences between cities.

The TOD improvement (renewal) project around the metro station differs from the general TOD evaluation. Smooth progress of the project needs to incorporate the opinions of the residents. Similar views have also been expressed in related studies [20, 23, 25]. Therefore, it is necessary to conduct a survey of metro and non-metro passengers within the metro coverage area to get their opinions on the TOD improvement. The following four indicators are the main factors for the public opinion: residents’ daily living and travel locations, commercial consumption areas, metro passengers’ willingness to transform, and non-metro passengers’ willingness to take the metro.

The above indicators are classified according to the T/O/D attributes, where two types of rail transit indexes and their connection are classified as the T (transit) attribute, and the two types of indicators of residents’ willingness and the development potential around the station as the O (oriented) attribute. Density, land use, social services and economic development are also classified as D (development) attributes. Based on the TOD typology studies, the Ningbo TOD improvement project evaluation framework (see Table 1) is constructed as previously described. Details are presented later in Chapter 3.6.

### Three New Variables

The three indicators introduced for the construction of the index system are as follows:

- Convenience of connection index (CCI, indicator 7)

CCI describes the convenience of connection for actual metro passengers at each station and describes the weighted average connection distance from the inverse sequence. The closer the connection distance, the higher the CCI and the better the connection convenience, and vice versa. CCI is defined as

1 $$CCI_{i} = 2000 - \frac{{\mathop \sum \nolimits_{{k = 1}}^{3} N_{{ik}} S_{k} }}{{\mathop \sum \nolimits_{{k = 1}}^{3} N_{{ik}} }},$$

where i is the index of the station number between 1 and 63, k is the connecting distance circle layer number (within 500 m: k = 1, 500–1000 m: k = 2, outside 1500 m: k = 3), N_ik_ in the questionnaire is the number of passengers who took the metro at the i-th station with the connecting distance at the k-th circle, S_k_ is the theoretical average distance of the k-th circle layer (within 500 m: 250, within 500–1000 m: 750, outside 1500 m: taken as 2000).

(b) (b) Land-use diversity (the entropy of land properties, indicator 31)

This term gives the land entropy value according to the general formula while considering that some land-use properties in urban TOD development may reduce transit use. It treats the land of these properties as a negative value.

The entropy of land mix degree of the i-th station is

2 $$E = - \mathop \sum \limits_{{j = 1}}^{N} \left( {Q_{{ij}} \times \ln Q_{{ij}} } \right)/\ln N,$$

where Q_ij_ is the percentage of the land of type j at the i-th station in the total land surrounding the station, and N is the total number of land types around all stations.

(c) The rationality index of the circle development (Standard Gradient Index [SGI], indicator 32)

This term describes the step-change relationship of the floor area ratio of the three circles with a radius of 250 m, 500 m, and 1000 m. A standardized unit circle area range is used to describe the difference in floor area ratio. The development of the three circles is approximately close to the step distribution. The higher the SGI, the more reasonable the development of the circle, and vice versa.

3 $$SGI_{i} = {\text{ }}1/\left( {1 + \exp \left( { - G_{i} } \right)} \right),$$

where

$$\begin{aligned} & Gi{\text{ }} = {\text{ }}\left( {1/\pi } \right){\text{ }} \times {\text{ }}\left[ { - \left( {F_{{500}} - F_{{250}} } \right)/\left( {500^{2} - 250^{2} } \right) - \left( {F_{{1000}} - F_{{500}} } \right)/\left( {1000^{2} - 500^{2} } \right)} \right]{\text{ }} \times 10^{6} ~~\\ & F_{{250}} ,{\text{ }}F_{{500}} ,{\text{ }}F_{{1000}} :{\text{ }}250\;{\text{m}},500\;{\text{m}},1000\;{\text{m}}\;{\text{ range}}\;{\text{ Floor}}\;{\text{ Area}}\;{\text{ Ratio}}\;{\text{ }}\left( {{\text{FAR}}} \right) \\ \end{aligned}$$

## Case Study

**Fig. 3:**
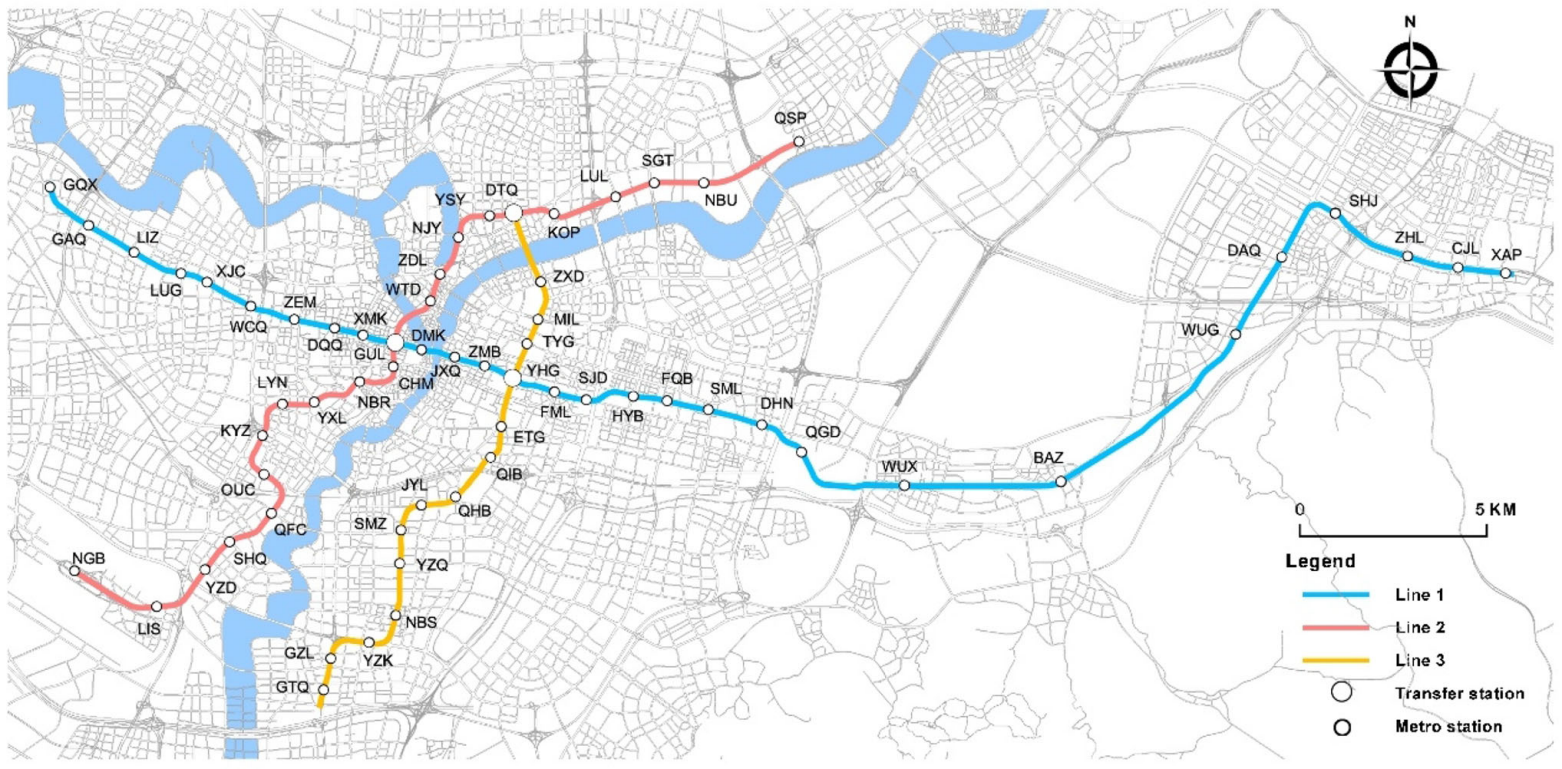
Ningbo metro network map (Oct. 2019)

The goal of this research is to identify which Ningbo metro stations are suitable for TOD improvement and optimization. Therefore, the defined research area includes all 63 stations covered by the Ningbo metro (see Fig. 3, as of Oct. 2019).

Ningbo is a city on the east coast of China, which is in the south wing of the Yangtze River Delta, with Shanghai as the Yangtze River Delta area centre. The current population of Ningbo city is six million, including three million residing in the urban areas and three million residing in the suburban areas [55].

### Dataset for Classification

The research data in this paper were obtained from the following sources:

- The inbound and outbound passenger flows were collected from the card data of the operating company.
- Data related to the traffic conditions around metro stations and connected passengers were provided by the Ningbo Transportation Planning Institute.
- Data related to the residents’ willingness were collected using surveys on mobile phone applications.
- Data related to on-site facilities were also collected using field surveys.
- The location of surrounding social service facilities was taken from GIS data and includes the density of convenience stores and density of medical and health facilities.
- The economic data were collected using the survey conducted by a professional agency.

Residents’ preferences were included as part of the project goal to renovate the periphery of the metro stations based on TOD [20, 23]. The passenger flow characteristics of the relatively new Ningbo metro system differ from those of cities with mature metro systems, e.g., Beijing and Tokyo. The inbound and outbound passenger flows from the station do not conform to the power-law distribution nor the log-normal distribution [56–58], and the number of inbound and outbound passengers is also not related to employment around the station. It is necessary, therefore, to supplement the questionnaires of residents (including metro passengers and non-metro passengers) and establish a connection between the number of questionnaires and the traffic, geographic, economic and other factors affecting metro ridership. This establishes a connection between the station volumes so that all the relevant data of the three attributes of T/O/D are integrated, which can be used as the basis of TOD improvement typology.

We carried out this research survey for the entire Ningbo city during January 2020, just before the Chinese New Year and COVID-19 outbreak. Sixty-three stations covered by the metro were surveyed, with 3087 valid samples obtained from 57 stations representing 1% of the passenger volume.

### Data Processing and Classification of Metro Stations

**Fig. 4:**
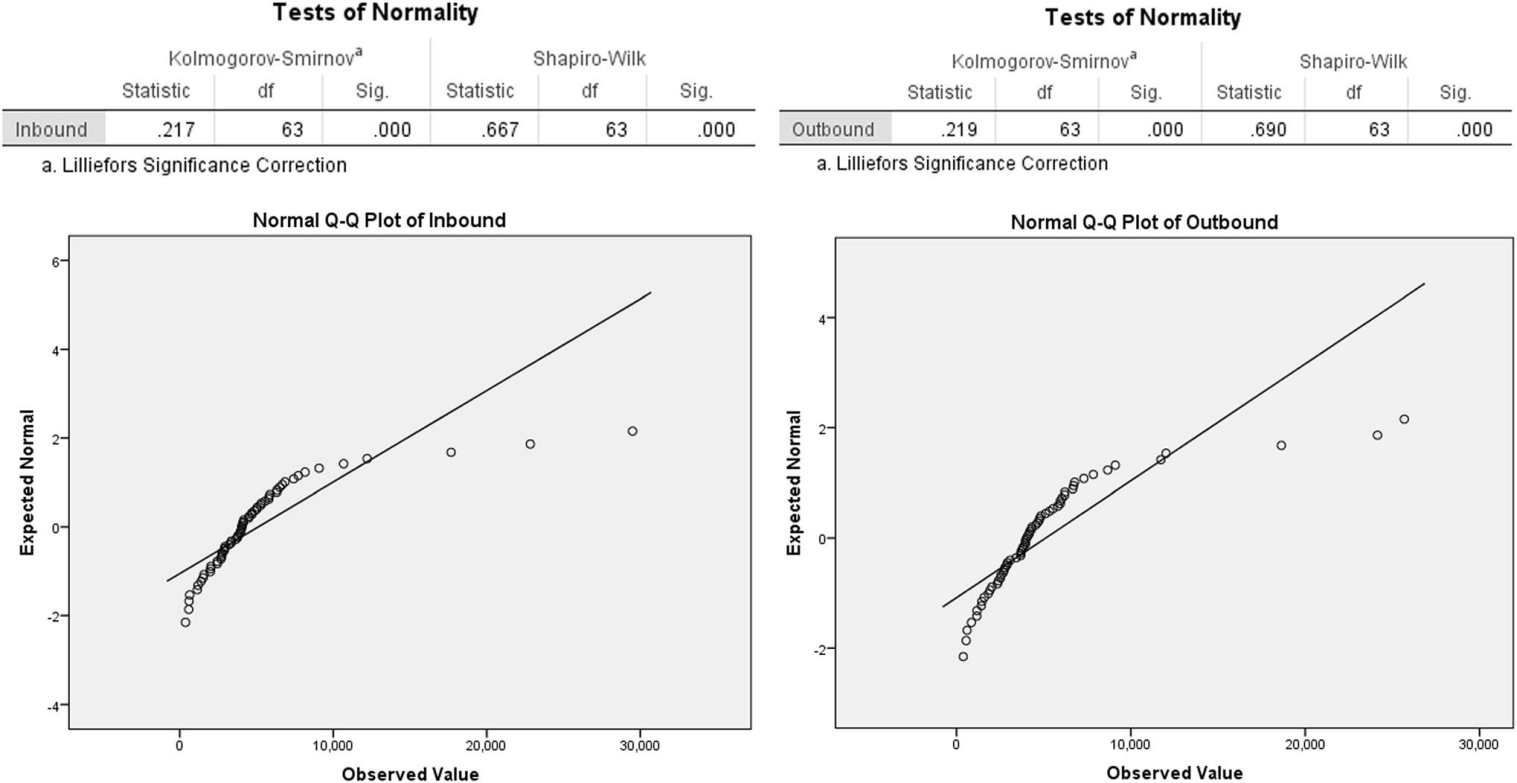
Normality test of passenger flow distribution in and out of the station

If the Ningbo metro had entered a mature development stage, and the passenger flow and surrounding land were relatively stable, then the TOD improvement methods of existing stations could be leveraged. Otherwise, the TOD development situations and improvement methods of stations in better-developed and well-known cities at home and abroad should be targeted instead. According to the studies on Beijing, Shanghai, Seoul, etc. [36, 59, 60], in East Asian cities with more mature metro development, the passenger flow in and out of each station shows a power-law or log-normal distribution. Passenger flow in and out of the station is correlated with the development of surrounding land, the number of surrounding populations, and the number of surrounding jobs. For Ningbo, however, the inbound and outbound passenger flows do not follow a power-law or log-normal distribution (see Table 2, Fig. 4). Furthermore, the passenger flow in and out of the station is not strictly related to the population and jobs distribution in radii of 250 m, 500 m, and 1000 m. This suggests that the development level of Ningbo metro TOD is still in its infancy (Table 3).

**Table 2:** Inbound and outbound passenger flow distribution test

Equation	Inbound R-square	Outbound R-square
Logarithmic	.864	.880
Quadratic	.680	.711
Cubic	.826	.851
Compound	.859	.863
Power	.769	.772
S	.400	.389
Exponential	.859	.863

**Table 3:** Inbound and outbound passenger flow correlation test

Spearman’s rho			Population250	Population500	Population1000	Job250	Job500	Job1000	FAR250	FAR500	FAR1000
(Indicator no.)			22	23	24	25	26	27	28	29	30
*Inbound*											
Correlation coefficient			.180	.185	.226	.348**	.468**	.565**	.157	.449**	.470**
Sig. (2-tailed)			.157	.147	.075	.005	.000	.000	.219	.000	.000
*Outbound*											
Correlation coefficient			.194	.190	.234	.360**	.473**	.572**	.160	.445**	.475**
Sig. (two-tailed)			.127	.136	.064	.004	.000	.000	.210	.000	.000

** Correlation is significant at the 0.01 level (2-tailed)

Through public surveys, it is found that the effective sample size of each station and the inbound and outbound passenger flow, the sample size and the planned population and jobs show different degrees of correlation (Spearman correlation [59]). This shows that the obtained sample is representative of the current passenger flow and the distribution of the surrounding population post. Survey data can be also used for TOD model data analysis (see Table 4)

**Table 4:** Effective sample size correlation test

Spearman's rho	Population250	Population500	Population1000	Job250	Job500	Job1000	FAR250	FAR500	FAR1000
(Indicator no.)	22	23	24	25	26	27	28	29	30
Correlation coefficient	.460**	.517**	.530**	.334**	.409**	.450**	.228	.503**	.531**
Sig. (two-tailed)	.000	.000	.000	.007	.001	.000	.073	.000	.000

** Correlation is significant at the 0.01 level (2-tailed)

**Fig. 5:**
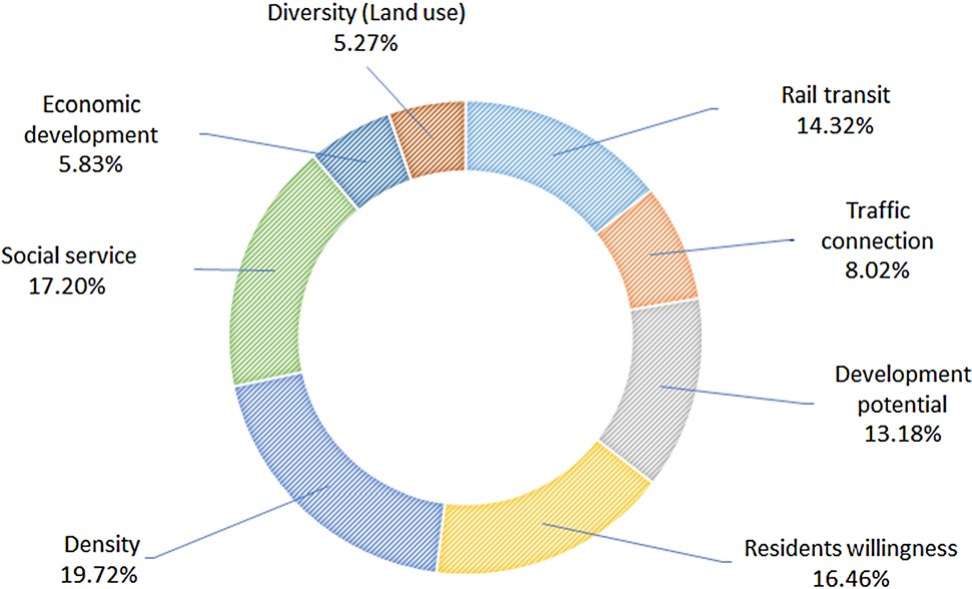
Eight criteria of influencing factors weight (by entropy calculation)

**Fig. 6:**
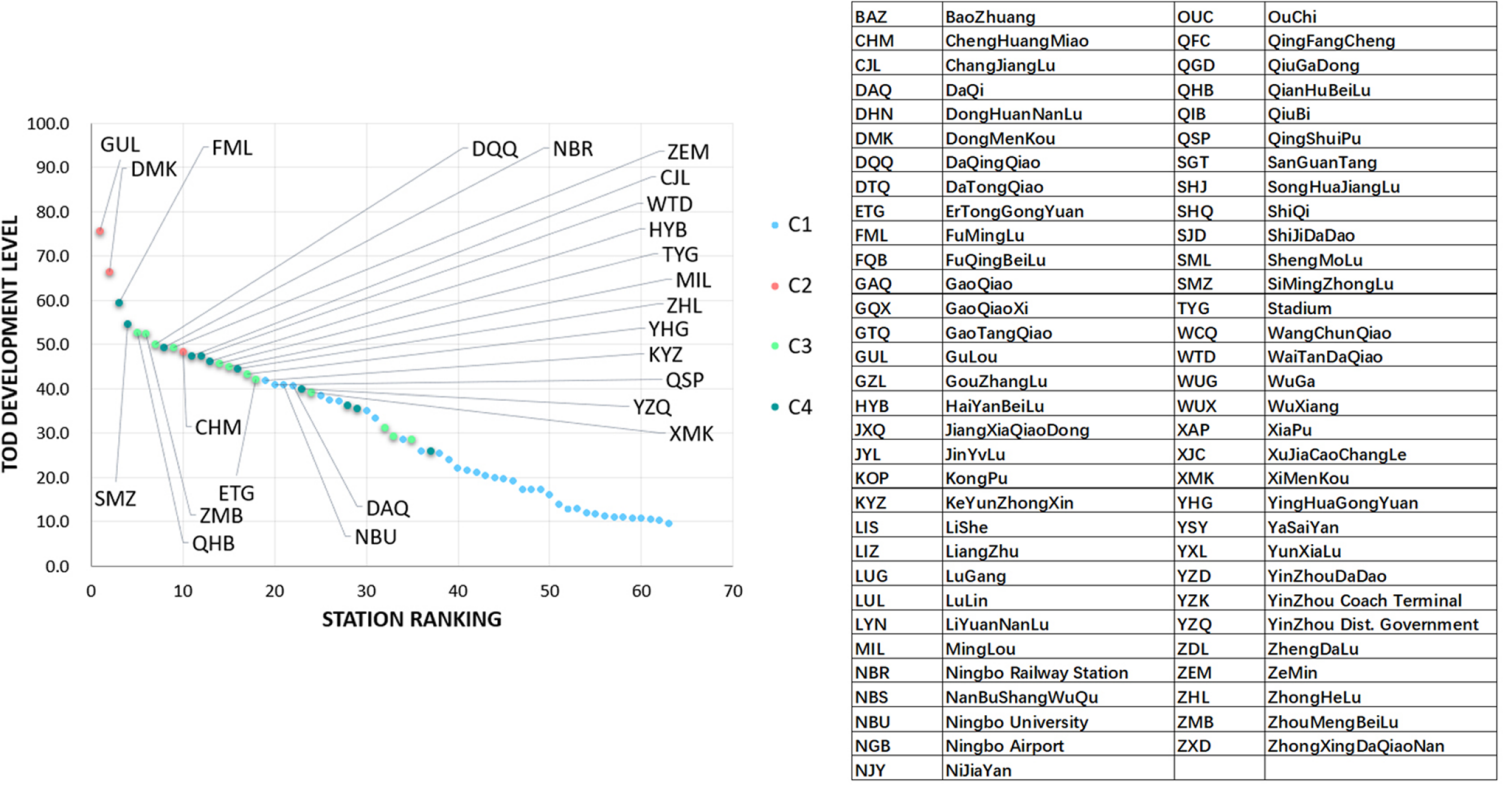
The TOD development score ranking of the station in Ningbo metro, 2019 (take the golden ratio of all the stations to mark, as it reflects a certain level of development, and is suitable as a candidate set for selecting TOD improvement)

The collected data are tested to determine whether the Cronbach-α coefficient is greater than 0.6, which is the basic condition for data reliability and variable selection. After testing, the Cronbach-α coefficient of all 39 variables is found to be greater than 0.6, and therefore the data are considered reliable. The data validity test is based on the Kaiser–Meyer–Olkin (KMO) value and Bartlett sphericity test. The KMO value of the test data is 0.737>0.6, and the *p*-value of the Bartlett sphericity test is <0.01. These also show that the selected 39 indicators can explain the evaluation of TOD improvement at stations. The entropy calculation weights are shown in Fig. 5. The evaluation of the TOD development level based on E-TOPSIS is also shown in Fig. 6.

**Fig. 7:**
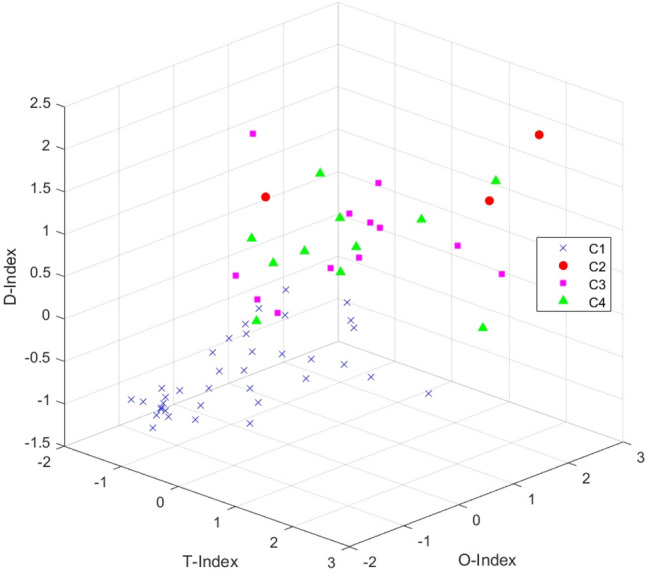
A 3D node-place model based on TOD improvement, including scatter plots of all 63 stations and four clusters

**Fig. 8:**
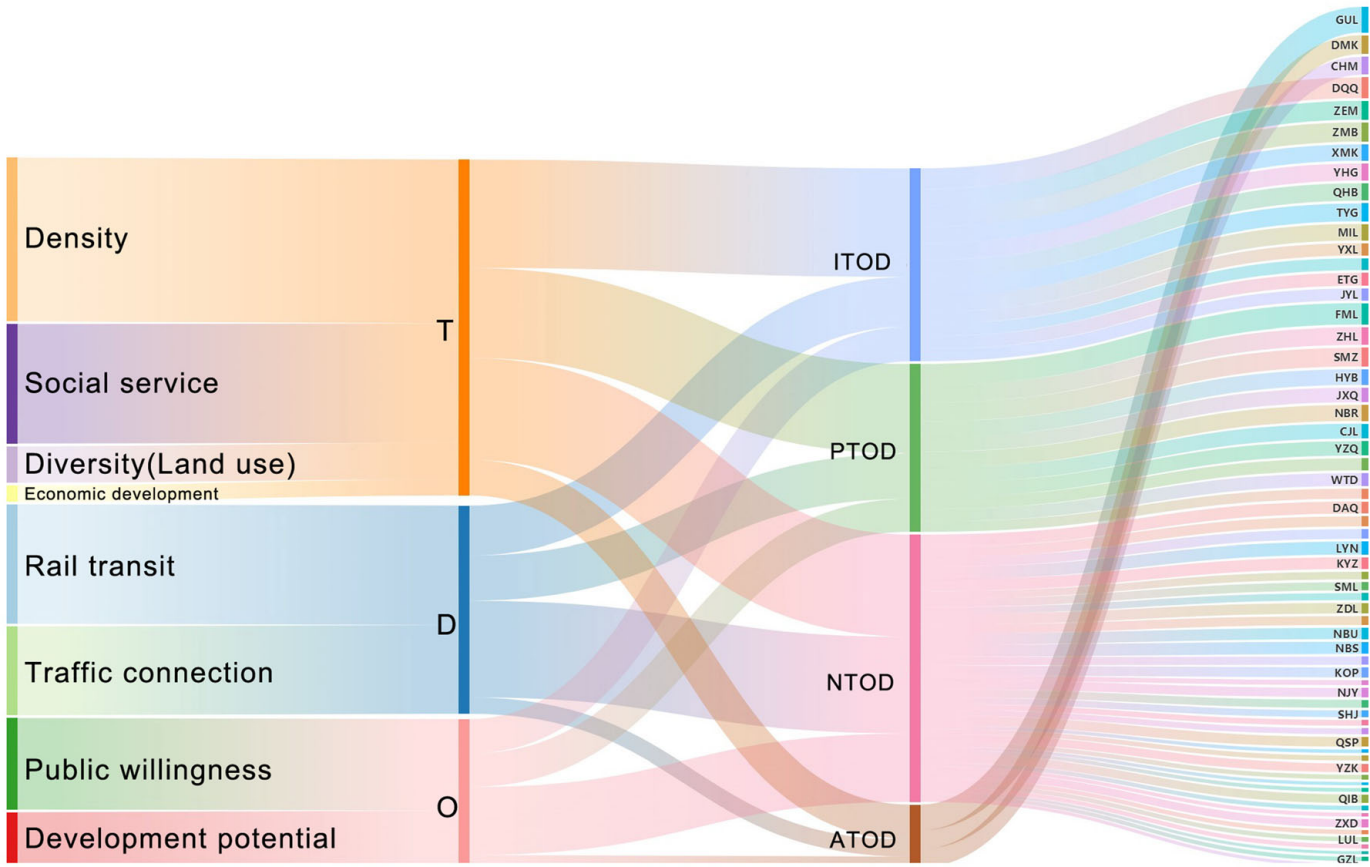
Sankey diagram: 39 indicators, 4 clusters, 63 stations

**Fig. 9:**
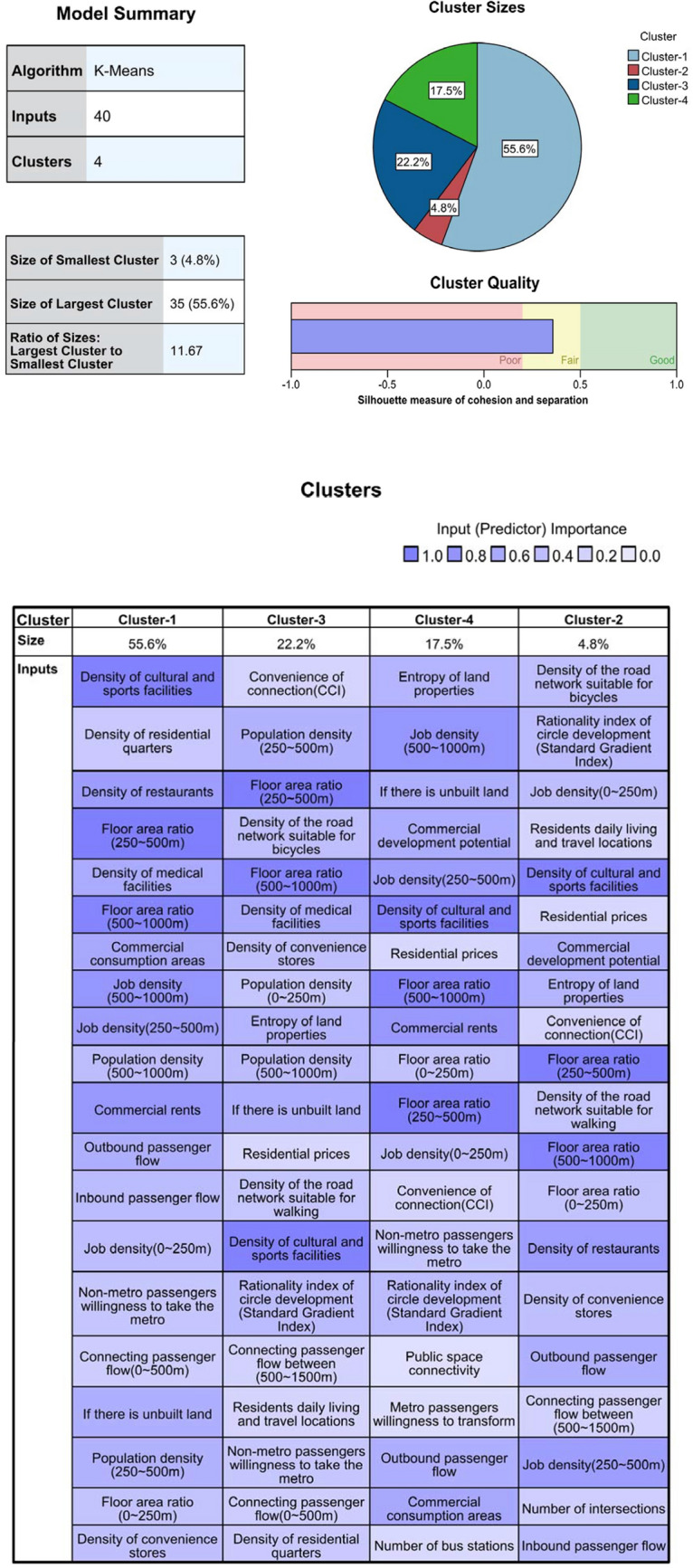
K-means cluster analysis results and top 10 within-cluster influencing factors heat map

It is shown that the use of machine learning methods [K-means [39, 40], self-organizing map (SOM) [29, 60], support vector machine (SVM), etc.) to classify TOD types helps reduce the interference of subjective factors in making a judgment. According to the T/O/D attribute characteristics of different indicators, they are included in the three types of attribute indicators. Based on the calculation of entropy weights, the T/O/D attribute score of each station is obtained, and then the result was calculated using the K-means cluster analysis. In the case of Ningbo, we found that the interpretability of K-means results is better than SOM and SVM. After applying z to transform the result, the visual output according to the T/O/D three-dimensional coordinates is presented in Figs. 7, 8, and 9.

## Results

### Four Types of Clusters

**Fig. 10:**
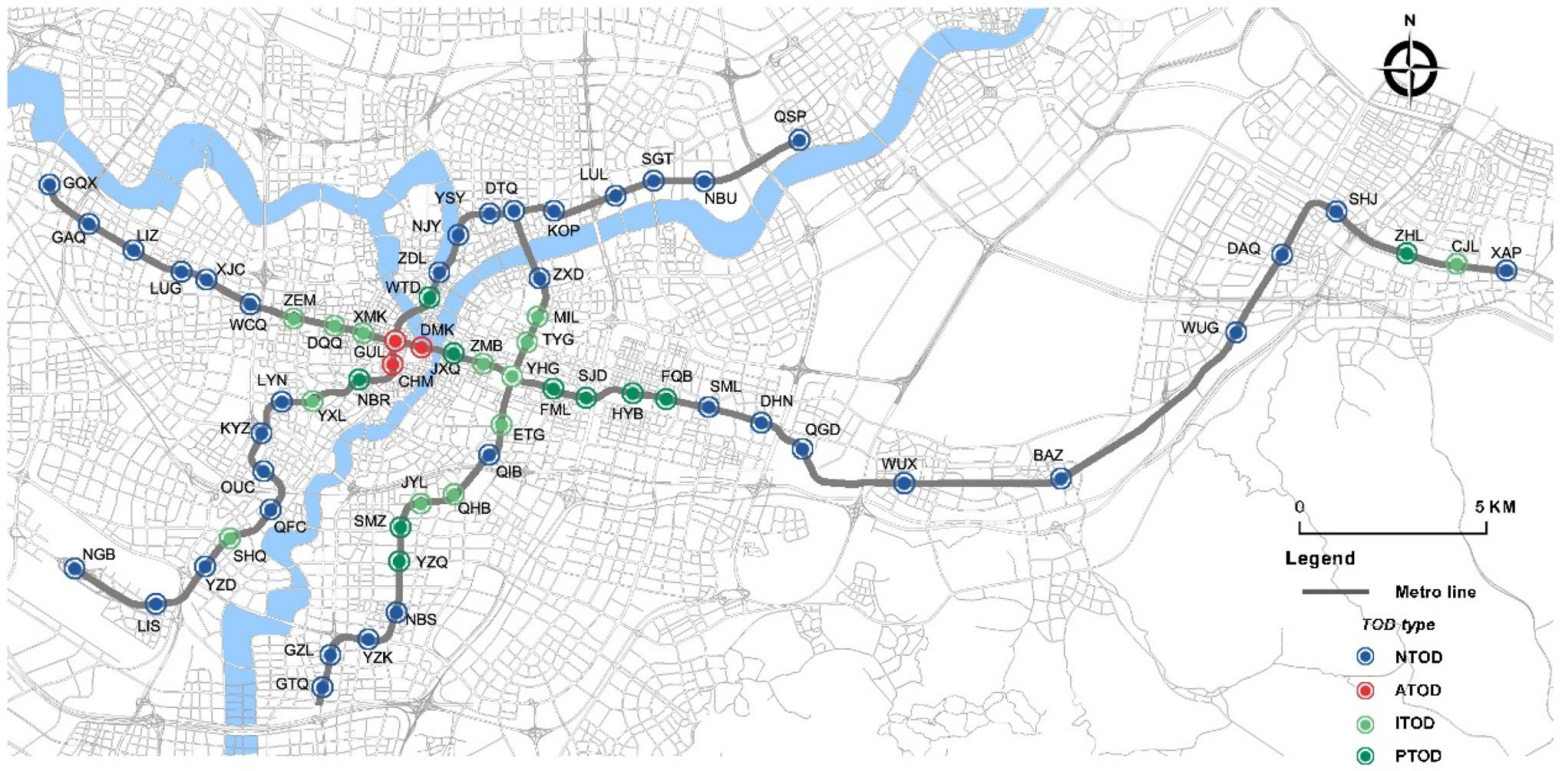
The classification of metro station areas in Ningbo City is based on the K-means analysis for 63 metro station areas

Based on the results of K-means clustering, the TOD improvement of Ningbo metro stations is divided into four clusters (see Fig. 10):

Cluster 1: NTOD (no TOD), has not yet shown a station with TOD significance, but a station with a better foundation may be transformed into a regional centre of the city.

Cluster 2: ATOD (already TOD)/city activities zone (CAZ), has developed a certain TOD and has become the core station of the city.

Cluster 3: ITOD (improvable TOD). The basic conditions for TOD development such as population and job density are reasonable, but there are shortcomings in at least one aspect of transportation supply, just like transportation connection, or economic development, which has not shown the TOD effect (rail transit has a large passenger flow and a high degree of urban mixing). Such stations are often in the construction region and have a large room for renovation and improvement, and are the key type recommended for renovation.

Cluster 4: PTOD (potential TOD), population and job density and other conditions are also good. There is no obvious shortcoming in the scoring performance. However, compared with station C1, it is not a city or district centre.

The distribution of the four types of stations in the Ningbo metro network is illustrated in Fig. 10.

### Target Station Recommendations Based on the Clustering Results

According to the node-place model based on the TOD improvement, the recommended station as the target of TOD improvement is the station near the four arcs enclosing the accessibility area. As the Ningbo metro is still in the initial construction stage, there is no station with a strong T-attribute (corresponding to the node attribute). Therefore, no station enters the U-N area, and the CAZ of Ningbo belongs to the CBD before the construction of the metro. During the same period, several regional commercial centres have emerged which have relatively weakened the position of the CAZ in the city’s commercial distribution. Therefore, no stations have entered the pressure area (S area).

**Fig. 11:**
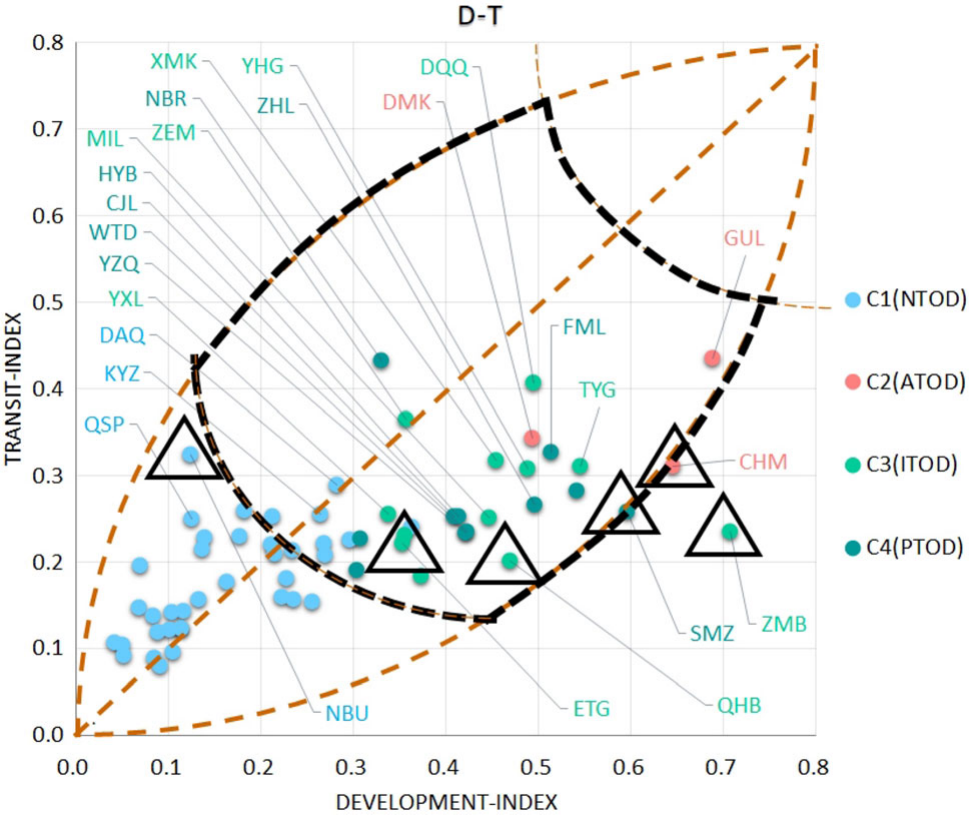
Node-place model for the TOD improvement and target station labelling (based on Fig 7)

**Fig. 12:**
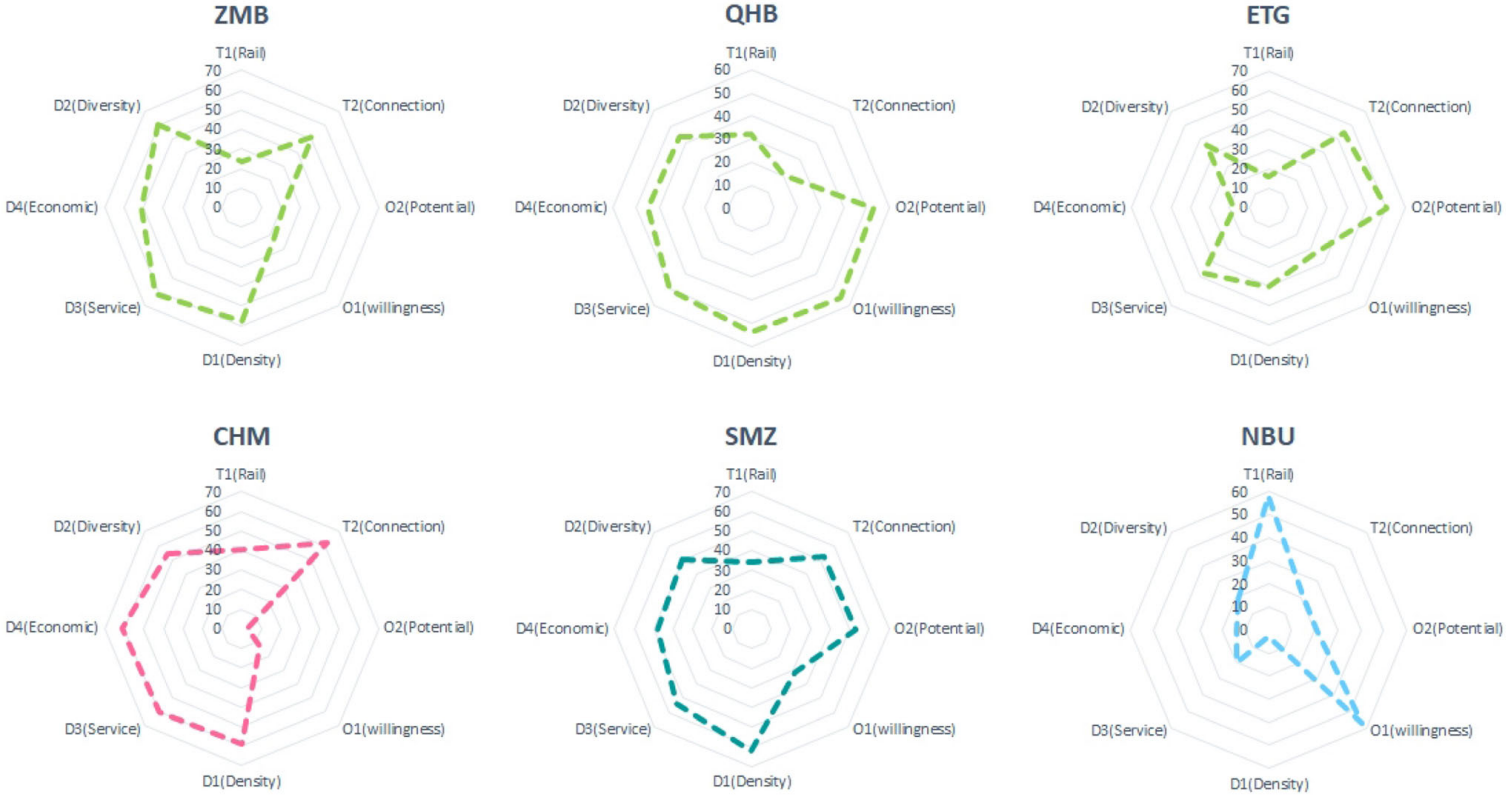
Radar chart of the typical stations

There are also no stations on the upper left and upper right edges of the Ac area. On the contrary, near the lower left and lower right edges, there are some stations with a certain development foundation (stations ranked in the front golden ratio in Fig. 6) close to these two curves. Considering the actual local situation, the following stations have also become our recommended stations (see Figs. 11 and 12):

1. Ningbo University (NBU) Station;

Stations that could be representative of TOD development value are not shown currently, and they are often located at the end of the line away from the traditional core in the dependency area on the distribution map based on type. The NBU station mainly benefits from the location, which is outside of the left arc, and is more likely to be attentively used because of the site, the connection conditions, social service density, and region of the surrounding residential area due to the existence of the university. Diversity and economic vitality are improved, more prone to improvement effects.

C2: ChengHuangMiao (CHM, The City God Temple) Station;

CHM station is one of three stations classified as ATOD. Compared to Beijing, Shanghai and other international metropolises, the CAZ region of Ningbo is not as developed. Three ATOD stations have not entered the stress area. Among them, the CHM station reflects their prominent place characteristics, such as commercial diversity and convenience, and support networks that are fully linked to the surroundings. Adding a metro to this site will attract riders, but the relationship between any improvements and complex regional characteristics requires more exploration.

C3: ZhouMengBeiLu (ZMB) Station, ErTongGongYuan (ETG, Children's Park) Station, QianHuBeiLu (QHB) Station;

QHB station has been developing along with more mature business districts in the city. The surrounding population continues to grow, the development index is high, and the transit index is low, mainly in terms of the low efficiency of rail transit and poor connectivity. As a more typical construction zone in the ITOD class, the next step should focus on improving the traffic environment. The ETG station represents the area under construction in the ITOD. Building construction and population growth are closely related to the level of economic development around the station. It is the key to the balanced state of TOD development.

C4: SiMingZhongLu (SMZ) Station;

This station is classified as a PTOD station, which belongs to the edge of the accessibility area. Located at the edge of the urban core area, there is still a certain gap between the development level and ATOD. As can be seen from Figs. 11 and 12, the indicators of the SMZ dimensions are partly within CHM, O2 criteria, indicating that the station still has further room for construction improvements related to both the facility itself and station accessibility.

## Discussion

Compared with cities with a long history of rail transit, the TOD level of metro stations in Ningbo seems more scattered [14, 26, 27, 39]. This shows that Ningbo’s TOD development level is not as high as that of cities where the rail transit has been opened to traffic for a long time.

In the node-place coordinate map, the overall distribution is biased towards the D-attribute axis (place attribute). This indicates that the stations that have developed into the centre lack transit guidance and there are still many stations that have been opened to transit but have not been yet developed.

No station enters the upper right stress area, implying that for most regional centres, the level of transit service and regional development are still insufficient. But the overall score of the existing commercial districts is higher than that of the residential districts. For instance, the transformation effect of Dong-gu Road (an underground commercial street connecting Gulou and Dongmenkou stations, and the whole is above the metro tunnel of Line 1) is very significant. Gulou and Dongmenkou stations that connect the two ends of Dong-gu road, rank the first two amongst all 63 stations.

Based on the analysis of the TOD typology clustering and radar chart analysis, the stations can be developed to allow diversified choices for different types of stations. After all, even the same type of station also may has different advantages and shortcomings and the selection of a specific transformation strategy should be based on the specific conditions of the station.

## Conclusions

The purpose of this research is to explore a set of TOD typology methods for developing cities represented by Ningbo. This is to describe the method of TOD improvement station selection based on metro stations and to explore the improvement direction. Moreover, based on the application of the traditional node-place model, the O-attribute is expanded according to the needs of the improvement project. The relevant indicators of the T- and D-attributes are extended according to the locally available data and the habitual TOD principle description. The model also introduces a machine learning method to determine that the station selection method is more valuable, easier to operate, and less disturbed by human factors. Whether specific indicators apply in other cities, and certain land uses have different TOD improvement values in other cities remains to be verified. Such research questions can be resolved in future research and practice. Whether this method can be combined with the traffic behaviour selection analysis and land-use adjustment strategies in the existing literature is also a direction worth exploring.

We have also identified four categories of tendencies for TOD improvement of metro stations in Ningbo. Although the basis of the research is the same as the traditional TOD-related research based on the node-place model, the article proposes a new direction for node-place model expansion for station area improvement. The presented expansion model can provide a reference for other cities that have recently built urban rail transit systems.
